# Hemoglobin is associated with cardiotoxicity in melanoma patients without anemia receiving immune checkpoint inhibitor therapy

**DOI:** 10.1016/j.ijcha.2025.101693

**Published:** 2025-05-08

**Authors:** Elias Haj-Yehia, Raluca I. Mincu, Phillip Schulte, Sebastian Korste, Samuel Dautzenberg, Lars Michel, Amir A. Mahabadi, Tienush Rassaf, Matthias Totzeck

**Affiliations:** Department of Cardiology and Vascular Medicine, West German Heart and Vascular Center Essen, University Hospital Essen, Essen, Germany

**Keywords:** Immune checkpoint inhibitor, Cancer therapy-related cardiovascular toxicity, Cancer therapy-related cardiac dysfunction, Hemoglobin, Melanoma

## Abstract

**Background:**

Low hemoglobin values are associated with cardiotoxicity in patients with melanoma and other cancer entities receiving immune checkpoint inhibitor (ICI) therapy. However, in cancer patients under chemotherapy, enhanced incidence of cardiotoxicity events are also reported with increasing hemoglobin values. So far, the association between hemoglobin values within the normal limits and the incidence of cardiotoxicity in melanoma patients treated with ICI therapy has not been examined.

**Methods:**

We analyzed 114 melanoma patients receiving ICI therapy (61 ± 13 years; 38 % female) from the prospective Essen Cardio-Oncology Registry (EcoR). Patients with cancer-related anemia (hemoglobin < 11 g/dL) were excluded from the analysis. Baseline hemoglobin levels were assessed at patient enrollment before initiation of ICI therapy. Endpoint was the whole spectrum of cancer therapy-related cardiovascular toxicity (CTR-CVT) according to the European guidelines on cardio-oncology with a median follow-up of 464 days.

**Results:**

Hemoglobin values and overall CTR-CVT were positively associated with hazard ratio (HR) rising in a J-shaped curve depending on increasing hemoglobin values. Subgroup analysis revealed only a significant association of hemoglobin and cancer therapy-related cardiac dysfunction (CTRCD) (HR: 1.417; 95 % confidence interval (CI): 1.101 – 1.825; *p* = 0.007). This association also remained significant after adjustment for further confounders.

**Conclusions:**

Hemoglobin values within the normal limits are associated with cardiovascular toxicity in terms of CTRCD in this cohort of melanoma patients receiving ICI treatment. Future studies are needed to investigate underlying mechanisms and validate the clinical utility of hemoglobin as a potential additional biomarker for risk stratification in cancer patients.

## Graphical abstract

**Figure:**
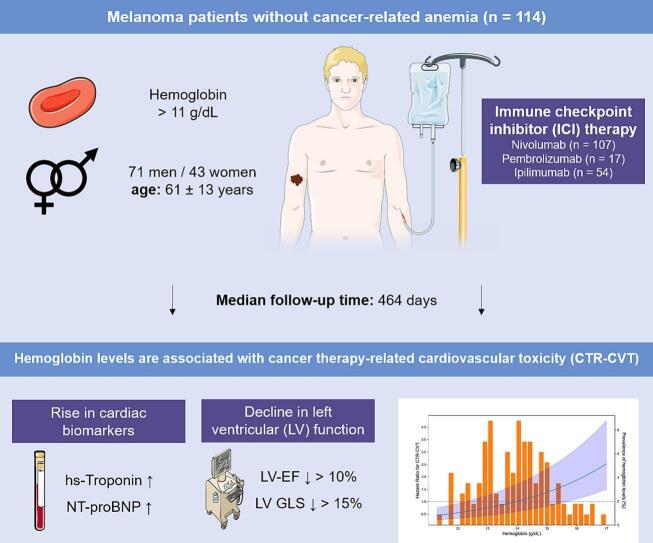

## 1 Introduction

Introduction of immune checkpoint inhibitor (ICI) therapy led to a significant improvement of mortality in melanoma patients [[1], [2], [3]]. These therapies induce an anti-tumor immune-reaction by blocking immune-inhibitory signaling [4,5]. Main targets are programmed cell death protein 1 (PD-1) and cytotoxic T lymphocyte-associated antigen-4 (CTLA-4) [5]. Among others, nivolumab and pembrolizumab are used as PD-1 targeting ICI therapy while ipilimumab inhibits interaction with CTLA-4 [5].

These therapies can lead to cardiotoxic side effects [[6], [7], [8]]. The European guidelines on cardio-oncology defined the whole spectrum of cancer-therapy related cardiovascular toxicity (CTR-CVT) [9]. Besides adverse events e.g., myocarditis, arrhythmia and vascular toxicity (e.g. myocardial infarction or stroke) also mild entities including cancer-therapy related cardiac dysfunction (CTRCD) characterized by increase in cardiac biomarkers (troponin or N-terminal prohormone of brain natriuretic peptide (NT-proBNP)) or decrease in left ventricular ejection fraction (LV-EF) and global longitudinal strain (LV GLS) are included [9].

Besides cardiovascular toxicity, ICI therapy can also lead to damage of erythrocytes and extracellular release of hemoglobin in form of hemolytic anemia as maximum expression [10,11]. Especially for melanoma patients, an increased risk of developing this hematologic toxicity is reported [12]. Under physiologic conditions, the main function of hemoglobin in red blood cells is the transport and delivery of oxygen [13]. However, cell-free hemoglobin in the circulation acts as a pro-oxidant and provokes pro-inflammatory reactions leading to tissue damage [11,14]. Some of these reactions, like release of pro-inflammatory cytokines, are also reported to contribute to ICI therapy-related cardiotoxicity [15].

As a biomarker, hemoglobin is mostly used for diagnosis of anemia [16]. In cancer patients, hemoglobin values below 11.0 g/dL are defined as cancer-related anemia according to the National Comprehensive Cancer Network (NCCN) guideline [16]. In these patients, a reduced treatment efficacy under ICI therapy and an increased rate of major adverse cardiac events (MACE) were observed [[17], [18], [19]]. Hemoglobin values ranging from 11.0 g/dL up to 18.0 g/dL are considered to be within the normal limits [20]. In cancer patients, only for breast cancer patients under chemotherapy a higher risk for cardiotoxicity is reported with increasing hemoglobin values [21]. While previous studies has investigated the cardiotoxic effects of ICI treatment and suggested a potential role for hemoglobin in this context, none have specifically focused on this association in melanoma patients. This study analyzed a well described cohort of melanoma patients receiving ICI therapy and aimed to evaluate the predictive capacity of hemoglobin values within the normal limits for CTR-CVT in these patients.

## 2 Methods

### 2.1 Study population

**Fig. 1:**
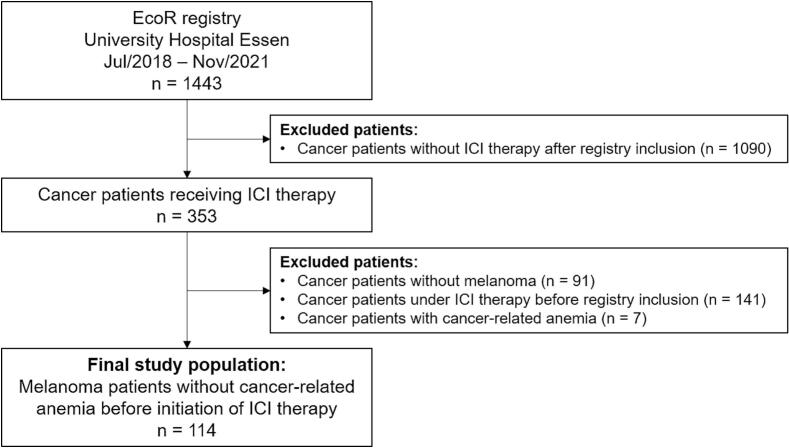
Flowchart of the study population included in the final analysis. Legend: Out of 1443 patients form the Essen cardio-oncology registry (EcoR) of the University Hospital Essen, 114 melanoma patients without cancer-related anemia before initiation of immune checkpoint inhibitor (ICI) therapy were identified. Excluded were 1090 patients not receiving ICI therapy, 91 patients without melanoma, 141 patients treated with ICI therapy before registry inclusion and 7 patients with cancer-related anemia.

The study collective was part of the Essen cardio-oncology registry (EcoR), a prospective registry enrolling patients evaluated in the local outpatient cardio-oncology unit at the University Hospital Essen. The registry was approved by the local ethics committee (19–8632-BO). It includes 1443 cancer patients, enrolled between July 2018 and November 2021, who provided written informed consent. In this study a subgroup of 114 melanoma patients without cancer-related anemia before initiation of ICI therapy were analyzed. Hemoglobin levels below 11 g/dL were defined as cancer-related anemia according to the NCCN guideline [16]. Exclusion criteria were no ICI therapy (1090 patients), patients without melanoma (91 patients), patients receiving ICI therapy before registry inclusion (141 patients), and existence of cancer-related anemia (7 patients) (Fig. 1). Patient enrollment and follow-up was performed by the local cardio-oncology outpatient unit. This included recording of baseline demographics, information about cancer-related characteristics and comorbidities, execution of clinical examination, electrocardiography (ECG) and echocardiography and obtaining of blood samples at patient enrollment. During follow-up, hemoglobin values were assessed after 6 weeks, 6 month and 1 year. Clinical examination, ECG, non-invasive cardiac imaging (echocardiography and cardiac magnetic resonance imaging (CMR)) and measurement of cardiac biomarkers (high-sensitive troponin and NT-proBNP) were used for assessment of CTR-CVT. Cancer therapy-related cardiac dysfunction (CTRCD), vascular toxicity, arrhythmia, new onset of arterial hypertension and myocarditis were defined as CTR-CVT according to the diagnosis criteria of the European guidelines on cardio-oncology [9]. CTRCD was defined as decline in LV GLS of more than 15 % or new rise in cardiac biomarkers (high-sensitive troponin or NT-proBNP) (mild CTRCD) or reduction of LV-EF of more than 10 % (moderate CTRCD). Vascular toxicity was defined as pulmonary embolism, deep venous thrombosis, myocardial infarction or stroke. Arrhythmia was defined as sinus tachycardia, sinus bradycardia or atrial fibrillation. Myocarditis was diagnosed as clinical diagnosis consisting of relevant troponin elevation in combination with positive CMR diagnostic for acute myocarditis and exclusion of coronary artery disease progression. The median follow-up was 464 days (interquartile range (IQR): 388–540 days).

### 2.2 Statistical analysis

Categorical variables were presented as absolute and relative frequencies (%) and association between these variables was evaluated by the use of Cochrane-Armitage test. Distribution of continuous variables was assessed using Kolmogorov-Smirnov test. Normally distributed continuous variables were expressed as mean ± standard deviation (SD) and non-normal distributed variables were displayed as median with IQR. Association between continuous variables and hemoglobin was assessed using Spearman correlation coefficient *ρ*. Kaplan-Meier cumulative event curves showed overall CTR-CVT in dependence of hemoglobin tertiles and were compared using log-rank test. Patients lost to follow-up were treated as censored observations. Median follow-up time was calculated by the reverse Kaplan-Meier method. The association between hemoglobin levels and CTR-CVT was analyzed using Cox proportional hazard model and univariable Cox regression. Different multivariable Cox regression models and backward elimination models were calculated to show the adjusted association of hemoglobin values and overall CTR-CVT or CTRCD. The predictive capacity of hemoglobin levels for CTRCD were computed using receiver operating characteristic (ROC) curve analysis. Youden’s index was used for optimal cut-off value determination. The performance of hemoglobin in the entire collective was compared to other clinically relevant biomarkers (NT-proBNP, high-sensitive troponin, C-reactive protein (CRP) and neutrophil-to-lymphocyte ratio (NLR)) by means of the area under the ROC curve (AUC) and statistically compared using DeLong’s test. The significance level was set as *p* < 0.05. As this study was an exploratory analysis, no further adjustments were made for multiple testing. All statistical analyses were performed using IBM SPSS 29.0 software (SPSS Inc.). Cochrane Armitage test, time-dependent ROC curve analysis and figures of ROC curve analysis and Kaplan-Meier cumulative event curves were generated with custom Python scripts using appropriate libraries. Statistical analysis for hemoglobin values during follow-up was conducted with Graphpad Prism 6 using ANOVA with Tukey correction for multiple comparisons or Mann-Whitney *U* test for single comparison.

## 3 Results

### 3.1 Baseline characteristics across hemoglobin tertiles

Hemoglobin values were assessed in a cohort of 114 melanoma patients (mean age 61 ± 13 years; 38 % female) at time of registry enrollment by the local cardio-oncology outpatient unit. Clinical and laboratory baseline characteristics of the study collective are depicted according to hemoglobin tertiles (low: < 13.3 g/dL; medium: 13.3 g/dL – 14.4 g/dL; high: > 14.4 g/dL) in Table 1. Despite female and older patients presenting with lower hemoglobin values, no differences concerning cancer disease, prior cancer treatment or cardiovascular comorbidities and medication were observed (Table 1). Hemoglobin values showed a maximum of 17.0 g/dL in this study collective (Fig. 3). Higher hemoglobin levels were associated with higher erythrocytes count and higher hematocrit, but displayed no differences in mean corpuscular volume (MCV), mean corpuscular hemoglobin (MCH) or count of platelets and leukocytes (Table 1). Hemoglobin values were not associated with kidney function or clinical relevant biomarkers like NT-proBNP, high-sensitive troponin, CRP or NLR (Table 1). Baseline ECG and echocardiography parameters showed no differences between patients separated by hemoglobin tertiles (Sup. Table S1). After registry enrollment ICI therapy was initiated in this analyzed subgroup of melanoma patients within a median of 5 days (IQR: 0 – 14 days; Table 2). The most favored ICI therapy was nivolumab (93.9 %) targeting PD-1 (Table 2). Median treatment duration was 149 days (IQR: 100 – 242 days; Table 2).

**Table 1:** Baseline characteristics.

		Tertiles of hemoglobin (g/dL)			
	Total (n = 114)	< 13.3 (n = 37)	13.3 – 14.4 (n = 40)	> 14.4 (n = 37)	*p*-value
Age (years), mean ± SD	61 ± 13	63 ± 13	62 ± 14	57 ± 11	0.039
Sex (female), n (%)	43 (37.7)	23 (62.2)	13 (32.5)	7 (18.9)	<0.001
BMI, median (IQR)	26.0 (24.0–30.7)	26.0 (22.7–30.6)	25.7 (24.8–29.1)	26.2 (23.8–32.9)	0.24
**Cancer disease and prior cancer treatment**					
Cancer manifestation before ICI treatment initiation (months),median (IQR)	28 (16–57)	28 (17–45)	34 (15–72)	22 (17–55)	0.60
Metastasis, n (%)	68 (59.6)	24 (64.9)	27 (67.5)	17 (45.9)	0.098
Surgery, n (%)	112 (98.2)	36 (97.3)	39 (97.5)	37 (100.0)	0.38
Radiation, n (%)	13 (11.4)	3 (8.1)	5 (12.5)	5 (13.5)	0.47
Chemotherapy, n (%)	14 (12.3)	6 (16.2)	5 (12.5)	3 (8.1)	0.29
B-Raf inhibitor, n(%)	9 (7.9)	3 (8.1)	3 (7.5)	3 (8.1)	1.00
MEK inhibitor, n (%)	9 (7.9)	3 (8.1)	3 (7.5)	3 (8.1)	1.00
Other, n (%)	5 (4.4)	3 (8.1)	2 (5.0)	0	0.089
**Cardiovascular Comorbidities, n (%)**					
Coronary artery disease	9 (7.9)	4 (10.8)	3 (7.5)	2 (5.4)	0.39
Atrial fibrillation	5 (4.4)	0	3 (7.5)	2 (5.4)	0.26
Previous stroke	9 (7.9)	3 (8.1)	4 (10.0)	2 (5.4)	0.67
Hypertension	56 (49.1)	19 (51.4)	22 (55.0)	15 (40.5)	0.35
Diabetes mellitus	8 (7.0)	2 (5.4)	4 (10.0)	2 (5.4)	1.00
Dyslipidemia	8 (7.0)	5 (13.5)	2 (5.0)	1 (2.7)	0.069
History of smoking (current or past)	18 (15.8)	5 (13.5)	5 (12.5)	8 (21.6)	0.34
Obesity	31 (27.2)	11 (29.7)	9 (22.5)	11 (29.7)	1.00
**Cardiovascular medication, n (%)**					
ASS	15 (13.2)	6 (16.2)	7 (17.5)	2 (5.4)	0.17
Statin	14 (12.3)	6 (16.2)	7 (17.5)	1 (2.7)	0.078
β blocker	24 (21.1)	7 (18.9)	9 (22.5)	8 (21.6)	0.78
ACEi or ARB	48 (42.1)	19 (51.4)	15 (37.5)	14 (37.8)	0.24
Oral anticoagulation	9 (7.9)	4 (10.8)	4 (10.0)	1 (2.7)	0.20
**Laboratory parameters**					
Hemoglobin (g/dL), mean ± SD	14.0 ± 1.2	12.6 ± 0.5	14.0 ± 0.3	15.3 ± 0.6	<0.001
Hematocrit (L/L), mean ± SD	0.41 ± 0.04	0.37 ± 0.02	0.41 ± 0.02	0.45 ± 0.02	<0.001
Erythrocytes (count/pL), mean ± SD	4.7 ± 0.5	4.3 ± 0.3	4.7 ± 0.3	5.1 ± 0.4	<0.001
MCV (fL), mean ± SD	88.0 ± 4.5	87.8 ± 4.2	88.4 ± 4.2	87.8 ± 5.2	0.73
MCH (pg), mean ± SD	29.9 ± 1.8	29.6 ± 1.8	29.8 ± 1.8	30.2 ± 1.8	0.39
Platelets (count/nL), median (IQR)	258 (218–298)	274 (232–322)	242 (212–293)	263 (220–289)	0.32
Leucocytes (count/nL), median (IQR)	6.6 (5.5–8.5)	6.5 (5.6–7.5)	6.5 (5.2–8.5)	6.8 (5.8–9.8)	0.13
eGFR CKD-EPI (mL/min/1.73 m^2^), mean ± SD	79.4 ± 15.9	76.9 ± 16.5	79.0 ± 16.8	82.3 ± 14.3	0.49
CRP (mg/dL), median (IQR)	1.0 (0.4–3.0)	0.8 (0.4–3.0)	1.5 (0.5–3.7)	0.9 (0.4–3.0)	0.49
NLR, median (IQR)	2.8 (2.0–4.0)	2.4 (2.0–3.8)	2.9 (2.0–4.7)	2.8 (2.0–3.6)	0.53
hsTrop (ng/L), median (IQR)	5.0 (3.0–10.0)	6.0 (3.0–12.0)	5.0 (3.0–9.0)	5.0 (3.0–11.0)	0.80
NT-proBNP (pg/mL), median (IQR)	332 (141–909)	267 (152–1733)	333 (131–843)	379 (119–966)	0.72

Continuous variables are expressed as mean ± standard deviation (SD) or median (interquartile range (IQR)) in case of non-normally distributed data. Categorical variables are shown as frequencies and percentages (%). BMI, body mass index; ICI, immune checkpoint inhibitor; B-Raf, B-rapidly accelerated fibrosarcoma; MEK, mitogen-activated protein kinase; ASS, acetylsalicylic acid; ACEi, angiotensin-converting enzyme inhibitor; ARB, angiotensin receptor blocker; MCV, mean corpuscular volume; MCH, mean corpuscular hemoglobin; eGFR CKD-EPI; estimated glomerular filtration rate using the formula of the Chronic Kidney Disease Epidemiology Collaboration; CRP, C-reactive protein; NLR, neutrophil-to-lymphocyte ratio; hsTrop, high-sensitive troponin; NT-proBNP, N-terminal prohormone of brain natriuretic peptide.

**Table 2:** Information on initiated immune checkpoint inhibitor (ICI) therapy.

		Tertiles of hemoglobin (g/dL)			
	Total (n = 114)	< 13.3 (n = 37)	13.3 – 14.4 (n = 40)	> 14.4 (n = 37)	*p*-value
Nivolumab (PD-1), n (%)	107 (93.9)	36 (97.3)	36 (90.0)	35 (94.6)	0.63
Total dose of nivolumab (mg), median (IQR)	1898 (480–4870)	2149 (360–5216)	1630 (480–3840)	1852 (765–5760)	0.26
Pembrolizumab (PD-1), n (%)	17 (14.9)	3 (8.1)	7 (17.5)	7 (18.9)	0.19
Total dose of pembrolizumab (mg), median (IQR)	1000 (450–1750)	800 (400–1800)	1200 (400–2000)	900 (550–1800)	0.97
Nivolumab + Pembrolizumab, n (%)	10 (8.8)	2 (5.4)	3 (7.5)	5 (13.5)	0.22
Ipilimumab (CTLA-4), n (%)	54 (47.4)	18 (48.6)	19 (47.5)	17 (45.9)	0.82
Total dose of ipilimumab (mg),median (IQR)	624 (398–1010)	485 (332–987)	618 (480–990)	750 (490–1118)	0.14
CTLA-4 + PD-1	54 (47.4)	18 (48.6)	19 (47.5)	17 (45.9)	0.82
Duration of ICI therapy (days),median (IQR)	149 (100–242)	187 (101–322)	105 (64–225)	151 (124–211)	0.91
Time to initiation of ICI therapy (days), median (IQR)	5 (0–14)	4 (0–14)	3 (0–11)	8 (1–18)	0.20

Continuous variables are expressed as median (interquartile range (IQR)). Categorical variables are shown as frequencies and percentages (%). PD-1, programmed cell death protein 1; CTLA-4, cytotoxic T lymphocyte-associated antigen-4.

### 3.2 Association of hemoglobin and cancer therapy-related cardiovascular toxicity

**Fig. 2:**
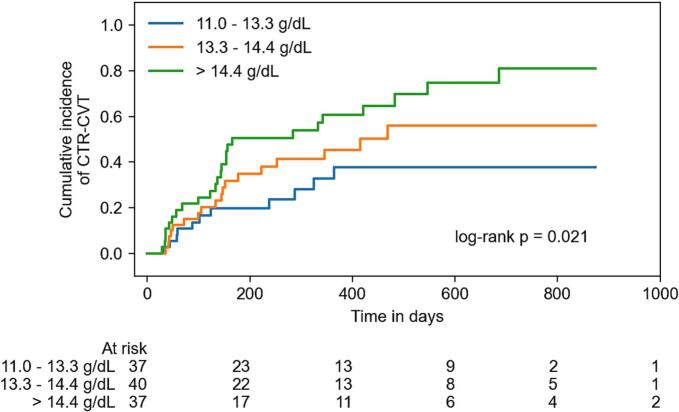
Cumulative incidence of overall cancer therapy-related cardiovascular toxicity (CTR-CVT) by hemoglobin tertiles. Legend: Kaplan-Meier cumulative event curves for overall cancer therapy-related cardiovascular toxicity (CTR-CVT) with melanoma patients separated by hemoglobin tertiles showed an increase in the cumulative incidence of CTR-CVT with rising hemoglobin baseline levels before initiation of immune checkpoint inhibitor (ICI) therapy.

**Fig. 3:**
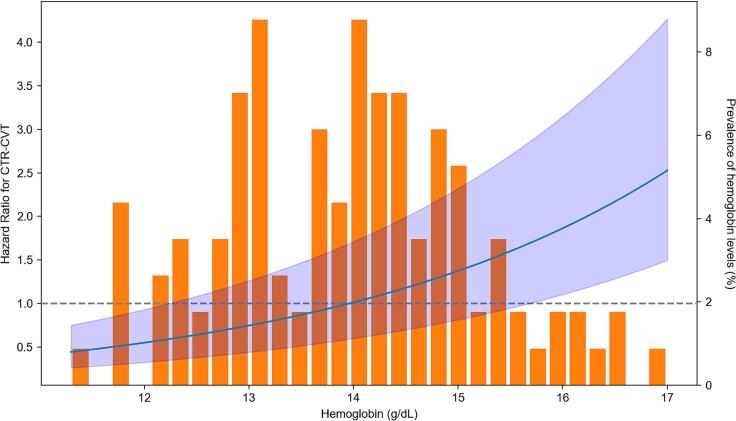
Cox proportional hazard model for overall cancer therapy-related cardiovascular toxicity (CTR-CVT) depending on hemoglobin values. Legend: Hazard ratio (blue line; with 95 % confidence interval (light blue area)) for overall cancer therapy-related cardiovascular toxicity (CTR-CVT) rises in a J-shaped curve depending on increasing hemoglobin values. Prevalence of hemoglobin levels for the entire study collective are depicted as orange bars and range from 11.3 g/dL up to 17.0 g/dL.

Out of all patients, CTR-CVT occurred in 54 patients (47.4 %) during follow-up (Table 3). Most of them developed CTRCD (41 patients (36.0 %)) followed by vascular toxicity and arrhythmia (12 patients (10.5 %) respectively) (Table 3, Sup. Table S2). More than one entity of CTR-CVT was observed in 18 patients (15.8 %). Higher hemoglobin values were associated with overall CTR-CVT as depicted for hemoglobin tertiles (log-rank *p* = 0.021; Fig. 2). Hazard ratio for CTR-CVT was enhanced in a J-shaped curve depending on increasing hemoglobin levels (Fig. 3). Overall survival was unaltered between hemoglobin tertiles (log-rank *p* = 0.236; Sup. Fig. S1A).

**Table 3:** Univariable Cox regression for hemoglobin.

Outcome	Number of events	Estimated hazard ratio (95 % CI)	*p*-value
Overall CTR-CVT	54	1.355 (1.088–1.687)	0.007
CTRCD	41	1.417 (1.101–1.825)	0.007
Vascular toxicity	12	1.369 (0.864–2.171)	0.18
Arrhythmia	12	1.105 (0.694–1.760)	0.67
New onset of arterial hypertension	5	1.483 (0.720–3.055)	0.29
Myocarditis	2	0.518 (0.139–1.929)	0.33
All-cause mortality	16	1.375 (0.892–2.123)	0.15

CI, confidence interval; CTR-CVT, cancer therapy-related cardiovascular toxicity; CTRCD, cancer therapy-related cardiovascular dysfunction.

Similar results were revealed by univariable Cox regression showing an association of hemoglobin with overall CTR-CVT in this study collective (hazard ratio (HR): 1.355; 95 % confidence interval (CI): 1.088 – 1.687; *p* = 0.007; Table 3). This was mainly attributable to a significant association of hemoglobin with CTRCD (HR: 1.417; 95 % CI: 1.101 – 1.825; *p* = 0.007; Table 3), whereby most of this patients developed mild CTRCD (39 patients (95.1 %); Sup. Table S3). Hemoglobin values were not associated with all-cause mortality in this study cohort (HR: 1.375; 95 % CI: 0.892–2.123; *p* = 0.151; Table 3). Association of hemoglobin with either overall CTR-CVT (HR: 1.366; 95 % CI: 1.069 – 1.746; *p* = 0.013) or CTRCD (HR: 1.435; 95 % CI: 1.077 – 1.913; *p* = 0.014) remained significant after adjustment for age and sex (Model 1; Table 4). Similar results were observed after additional adjustment for cardiovascular comorbidities (Model 2; overall CTR-CVT: HR: 1.389; 95 % CI: 1.071 – 1.801; *p* = 0.013; CTRCD: HR: 1.481; 95 % CI: 1.091 – 2.009; *p* = 0.012; Table 4). Further adjustment for cancer pre-treatment, metastasis status and duration of ICI therapy after registry enrollment led to a loss of significance for overall CTR-CVT (Model 3; HR: 1.302; 95 % CI: 0.067 – 1.729; *p* = 0.067; Table 4). However, the association of hemoglobin and CTRCD remained significant after this adjustment (Model 3; HR: 1.455; 95 % CI: 1.034 – 2.048; *p* = 0.032; Table 4). The association of hemoglobin with overall CTR-CVT (HR: 1.595; 95 % CI: 0.916 – 2.778; *p* = 0.099) was also not significant after additional adjustment for laboratory parameters, left ventricular ejection fraction (LV-EF) and left ventricular global longitudinal strain (LV GLS) but significance remained for the association of hemoglobin and CTRCD (HR: 2.162; 95 % CI: 1.077 – 4.341; *p* = 0.030) (Model 4; Table 4). Adjustment for cardiovascular medication did not affect the significant association of hemoglobin with overall CTR-CVT or CTRCD (Sup. Table S4). Furthermore, an additional Cox regression model was constructed using backward elimination with a significance level of 0.1 to select predictor variables for occurrence of overall CTR-CVT and CTRCD. The initial model included all variables used for Model 4 of the multivariable Cox regression depicted in Table 4. After stepwise elimination, hemoglobin remained significant for prediction of overall CTR-CVT (HR: 1.512; 95 % CI: 1.084–2.109; *p* = 0.015; Sup. Table S5) and CTRCD (HR: 1.909; 95 % CI: 1.182–3.082; *p* = 0.008; Sup. Table S5).

**Table 4:** Multivariable Cox Regression for hemoglobin.

Outcome	Model 1		Model 2		Model 3		Model 4	
	Estimated hazard ratio (95 % CI)	*p*-value	Estimated hazard ratio (95 % CI)	*p*-value	Estimated hazard ratio (95 % CI)	*p*-value	Estimated hazard ratio (95 % CI)	*p*-value
Overall CTR-CVT	1.366 (1.069–1.746)	0.013	1.389 (1.071–1.801)	0.013	1.302 (0.981–1.729)	0.067	1.595 (0.916–2.778)	0.099
CTRCD	1.435 (1.077–1.913)	0.014	1.481 (1.091–2.009)	0.012	1.455 (1.034–2.048)	0.032	2.162 (1.077–4.341)	0.030

**Model 1:** Adjustment for age and sex; **Model 2:** Adjustment for age, sex, coronary artery disease, atrial fibrillation, previous stroke, hypertension, diabetes mellitus, dyslipidemia, history of smoking and obesity; **Model 3:** Adjustment for age, sex, coronary artery disease, atrial fibrillation, previous stroke, hypertension, diabetes mellitus, dyslipidemia, history of smoking, obesity, metastasis status, duration of immune checkpoint inhibitor (ICI) therapy after registry enrollment, previous cancer surgery, previous cancer radiation and chemotherapy; **Model 4:** Adjustment for age, sex, coronary artery disease, atrial fibrillation, previous stroke, hypertension, diabetes mellitus, dyslipidemia, history of smoking, obesity, metastasis status, duration of immune checkpoint inhibitor (ICI) therapy after registry enrollment, previous cancer surgery, previous cancer radiation and chemotherapy, baseline left ventricular ejection fraction (LV-EF) and global longitudinal strain (LV GLS), leucocyte count, platelet count, NT-proBNP, high-sensitive troponin, CRP, NLR and eGFR. CI, confidence interval; CTR-CVT, cancer therapy-related cardiovascular toxicity; CTRCD, cancer therapy-related cardiac dysfunction.

### 3.3 ROC curve analysis for prediction of cancer therapy-related cardiac dysfunction

**Fig. 4:**
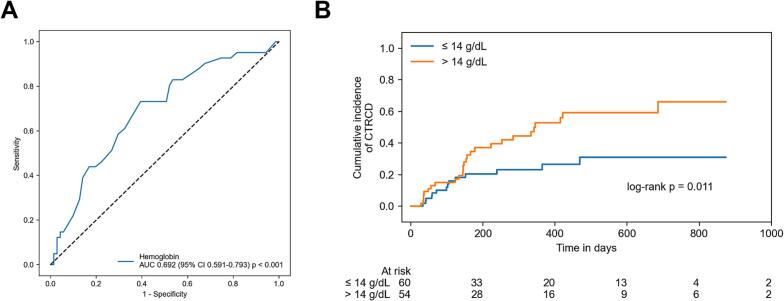
Receiver operator characteristics (ROC) analysis of hemoglobin for prediction of cancer therapy-related cardiac dysfunction (CTRCD). Legend: Receiver operator characteristic (ROC) curve analysis of hemoglobin for prediction of cancer therapy-related cardiac dysfunction (CTRCD) revealed an optimal cut-off value of 14.0 g/dL (A). Kaplan-Meier cumulative event curves for CTRCD with patients separated by low (≤ 14.0 g/dL) and high (> 14 g/dL) hemoglobin values according to ROC curve analysis showed higher incidence of CTRCD in patients with hemoglobin values > 14 g/dL (B). AUC, area under the curve; CI, confidence interval.

**Fig. 5:**
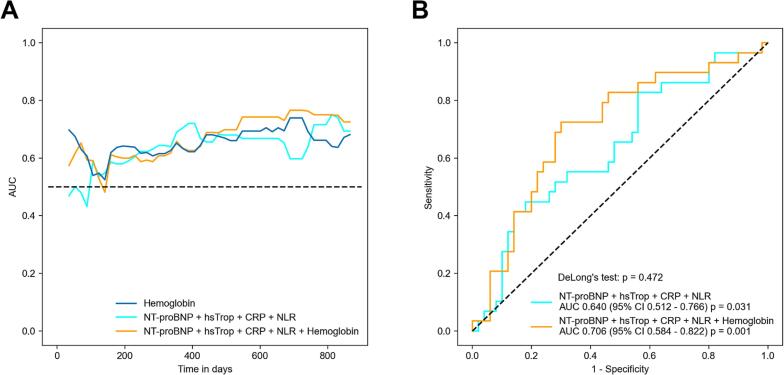
Addition of hemoglobin to established biomarkers for prediction of cancer therapy-related cardiac dysfunction (CTRCD). Time dependent receiver operator characteristic (ROC) curve analysis of hemoglobin and established biomarker (N-terminal prohormone of brain natriuretic peptide (NT-proBNP), high-sensitive troponin (hsTrop), C-reactive protein (CRP) and neutrophil-to-lymphocyte ratio (NLR)) with and without hemoglobin for prediction of cancer therapy-related cardiac dysfunction (CTRCD) showed the highest predictive capacity for long-term incidence of CTRCD (A). Addition of hemoglobin to NT-proBNP, high-sensitive Troponin, CRP and NLR led to an increase in area under the curve (AUC) at 2 years (B). CI, confidence interval.

ROC curve analysis highlighted the predictive capacity of hemoglobin for CTRCD in this study collective resulting in an AUC of 0.692 (95 % CI: 0.591 – 0.793; *p* < 0.001; Fig. 4A). The optimal cut-off value was 14.0 g/dL with 73.2 % sensitivity and 60.6 % specificity. Accordingly, 52 patients (45.6 %) presented with a hemoglobin value > 14.0 g/dL. These patients developed CTRCD significantly more often after initiation of ICI therapy (log-rank *p* = 0.011; Fig. 4B). Overall survival was not significant different in patients with hemoglobin values > 14.0 g/dL (log-rank *p* = 0.371; Sup. Fig. S1B). During follow-up, patients with baseline hemoglobin values ≤ 14.0 g/dL (13.05 g/dL ± 0.70 g/dL vs. 12.24 g/dL ± 1.57 g/dL; p = 0.02; Sup. Fig. 2A) and patients with hemoglobin values > 14.0 g/dL (14.95 g/dL ± 0.71 g/dL vs. 13.69 g/dL ± 2.18 g/dL; p < 0.001; Sup. Fig. S2A) showed a slight decrease of hemoglobin after 1 year. However, in patients with baseline hemoglobin values > 14.0 g/dL decrease of hemoglobin after 6 weeks was significant higher (−0.19 g/dL ± 0.98 g/dL vs. −0.55 g/dL ± 0.91 g/dL; p = 0.02; Sup. Fig. 2B). As Kaplan-Meier cumulative event curves for overall CTR-CVT and CTRCD (Fig. 2 and Fig. 4B) separated only after about 150 days of follow-up, we performed time-dependent ROC curve analysis. Accordingly, the lowest AUC values for prediction of CTRCD by hemoglobin were observed during the first 150 days followed by an increase in AUC at later time points (Fig. 5A). Further analysis showed that hemoglobin was not inferior compared to established biomarkers known to predict cardiotoxic events after ICI therapy (NT-proBNP, high-sensitive troponin, CRP and NLR) (Sup. Fig. S3) [[22], [23], [24], [25], [26]]. Addition of hemoglobin to these biomarkers led to an increase in AUC from 0.640 (NT-proBNP + high-sensitive troponin + CRP + NLR) to 0.706 (NT-proBNP + high-sensitive troponin + CRP + NLR + hemoglobin) at 2 years in this study collective (Fig. 5B). However, this AUC increase was not statistically significant (DeLong’s test *p* = 0.472; Fig. 5B).

## 4 Discussion

This study demonstrated that hemoglobin values within the normal limits are associated with cardiovascular toxicity in melanoma patients receiving ICI therapy. Subgroup analysis revealed only a significant association of hemoglobin and CTRCD, with the highest predictive capacity for late events. This association remained significant after adjustment for age and sex, cardiovascular comorbidities, cancer-related factors as well as laboratory and echocardiography parameters. Addition of hemoglobin to established biomarkers for CTRCD under ICI therapy did not significantly improved their predictive capacity.

Elevated hemoglobin levels are associated with increased mortality in long-term follow-up [27]. In a large cohort of non-cancer patients without cardiovascular disease hemoglobin levels were found to be positively associated with cardiovascular mortality [28]. Another study demonstrated elevated hemoglobin values being associated with the incidence of coronary heart disease and survival of patients with chronic heart failure [29,30]. Patients with increased pre-operative hemoglobin levels are at higher risk of in-hospital mortality after surgical coronary revascularization [31]. In breast cancer patients under chemotherapy hemoglobin was significantly associated with cardiotoxic reactions [21]. Our results demonstrating high hemoglobin values to be associated with CTRCD in melanoma patients without anemia under ICI therapy augment this evidence. Patients with high hemoglobin values might be particular endangered to develop “hidden cardiotoxicity” under ICI therapy as hemoglobin is also associated with cardiovascular comorbidities [32]. Our observation might not be transferrable to every type of cancer as a study cohort of mainly lung and digestive cancer patients did not show a significant association of hemoglobin with cardiotoxicity under ICI therapy [33]. Furthermore, a study investigating the factors accompanied with cardiotoxicity under ICI therapy in a heterogeneous cancer collective showed no differences in admission hemoglobin values for patients developing myocarditis [34].

ROC curve analysis of hemoglobin predicting CTRCD revealed an AUC of 0.692 in our study. This appears moderate for the prediction of cardiotoxicity [35]. However, compared to other studies in non-cancer patients investigating the association of hemoglobin with cardiovascular outcomes similar values for AUC are reported [[36], [37], [38]]. Nevertheless, in our study collective, hemoglobin performance was not inferior compared to established biomarkers for prediction of cardiotoxicity. As time-dependent ROC curve analysis revealed the best AUC values for hemoglobin after 150 days, utility for early risk stratification certainly is limited. Despite these aspects limiting the clinical utility of the calculated hemoglobin cut-off value of 14.0 g/dL for prediction of CTRCD in melanoma patients under ICI therapy in our study, these results provide first insights of an association between hemoglobin and the risk of cardiotoxicity in these patients. As this study provides detailed information about cancer history, ICI therapy regime, concomitant cardiovascular diseases as well as laboratory and echocardiography parameters, comprehensive analysis of additional confounding factors showed a robust and independent association of increased hemoglobin values with occurrence of cardiac dysfunction induced by ICI therapy in our study collective. However, larger randomized studies are needed in the future to achieve more valid hemoglobin thresholds with enhanced utility for clinical decision-making.

Our results suggest that higher values of hemoglobin might be driven by an increase in red blood cell count and hematocrit as levels of MCV and MCH remained unaltered. This is known to be associated with increased blood viscosity and shear stresses leading to enhanced hemolytic stress [39]. This provokes an increase in cell-free hemoglobin [40]. In addition, ICI therapy itself can cause damage of erythrocytes leading to enhanced extracellular release of hemoglobin [10]. Cell-free hemoglobin causes pro-oxidative effects, stimulates inflammatory response and can lead to tissue injury [11,14,41]. It is reported that circulating cell-free hemoglobin reduces the bioavailability of nitric oxide (NO) which might impairs its cardioprotective properties [[41], [42], [43], [44], [45]]. Furthermore, hemoglobin binds melanoma cell tissue factor and enhances its procoagulant activity which can augment further tissue damage [46]. These aspects could serve as an underlying explanation for the association of high hemoglobin values with CTRCD in this study collective. However, as our data do not provide direct measurement of cell-free hemoglobin, this explanation remains only speculative. Nevertheless, hemoglobin measurments during follow-up revealed a greater decrease at early time points in patients with higher baseline hemoglobin values, which might be associated with a greater extend of cell-free hemoglobin. On the other hand, these observations might be associated with other confounders like hemodilution effects, different treatment intensities or responses, bone marrow suppression from treatment as well as nutritional or inflammatory changes. Future studies should provide additional information concerning cell-free hemoglobin and inflammatory markers for more valid mechanistic examinations.

Concerning age and sex, hemoglobin values significantly differ. Older patients and women presented with lower hemoglobin values. This is rather unexpected as women exhibit lower hemoglobin values compared to men [47]. Among others, this is attributable for hormonal differences between both sexes, as androgens raise hemoglobin levels and estrogens lower them [48]. Studies also observed lower hemoglobin values in older patients as they suffer more often from iron deficiency or hematological disorders [49,50]. However, adjustment for age and sex did not affect the significant association of hemoglobin and overall CTR-CVT or CTRCD suggesting no age- or sex-specific differences for this biomarker in our study collective. In study collectives examine predictive capacity of hemoglobin in other diseases ambivalent observations for sex-specific differences were made. In a cohort of patients after myocardial infarction no sex-specific differences were observed for an association of hemoglobin with 12-month mortality [51]. Other studies however reported only in men a significant association of hemoglobin with cardiovascular risk and future events [52,53]. The female portion of melanoma patients in our study collective is rather small to permit a valid prediction for sex-specific differences. Therefore, larger trials are necessary to analyze this aspect more detailed.

## 5 Study limitations

Our study has several limitations. Cardiotoxic confounders like non-cardiovascular premedication apart from reported cancer treatment were not examined. There is also no information about time interval between previous cancer therapy, whether chemotherapy or radiation, and patient enrollment. Furthermore, no information distinguishing between hemoglobin associated with erythrocytes and cell-free hemoglobin is provided. We also analyzed a relatively small number of melanoma patients in an explorative manner. These patients are only from a single center. In addition, patients at higher risk developing cardiotoxicity under ICI therapy are more likely to attend to a local cardio-oncology outpatient unit. These aspects create a selection bias, which might be a confounder for incidence of CTR-CVT in this study. Furthermore, the analyzed melanoma patients received with nivolumab mostly one type of ICI therapy. In addition, non-melanoma cancer entities are not included in this analysis. Therefore, these results might not apply to other cancer entities or other types of ICI therapy. To address these limitations, larger prospective multi-center trials are needed investigating the association between hemoglobin values within normal limits and development of cardiotoxicity in other cancer entities treated with different types of ICI therapy.

## 6 Conclusion

Hemoglobin values within the normal limits were associated with overall CTR-CVT in melanoma patients without cancer-related anemia under ICI therapy. However, a subgroup analysis revealed that this applies only to CTRCD and the predictive capacity of hemoglobin in this study collective got lost after adjustment for further confounders. Furthermore, addition of hemoglobin to established biomarkers for prediction of CTRCD in cancer patients receiving ICI therapy did not significantly improved their performance. Nevertheless, our findings reveal first insights for an association of hemoglobin and cardiotoxicity in ICI-treated cancer patients. Future studies are needed to investigate underlying mechanisms and validate the clinical utility of hemoglobin as an additional biomarker for evaluation of cardiotoxic side effects in cancer patients under different types of ICI treatment.

Ethical approval

All procedures were in accordance with the ethical standards of the institutional ethics committees of the participating centers and with the 1964 Helsinki Declaration and its later amendments or comparable ethical standards.

**Availability of data and materials:** The dataset analyzed during the current study is available from the corresponding author upon reasonable request.*Sup.* Fig. S1*:*

**Disclosure:** E.H.-Y. reports personal fees and others from AstraZeneca, which are outside the submitted work. L.M. reports personal fees from Bayer, Alnylam, AstraZeneca, IFFM e. V. and from Bund der Niedergelassenen Kardiologen (BNK), which are outside the submitted work. M.T. and T.R. report personal fees and others from Edwards and Novartis, Bristol Myers Squibb, Bayer, Daiichi Sankyo and Astra Zeneca, which are outside the submitted work. T.R. cofounded Bimyo, a company focusing on the development of cardioprotective peptides. All other authors declare no conflict of interest.

**Address for correspondence:** Matthias Totzeck, Postal address: Department of Cardiology and Vascular Medicine, West German Heart and Vascular Center Essen, University Hospital Essen, Hufelandstraße 55, 45,147 Essen, Germany. matthias.totzeck@uk-essen.de. Telephone: +49 201 723 84805. Fax: +49 201 723 5401.

Funding

This work was supported by German Cardiac Society (DGK, Deutsche Gesellschaft für Kardiologie – Herz- und Kreislaufforschung e.V.) under Grant DGK02/2022 and Universitätsmedizin Essen Junior Clinical Scientist Academy (UMEA) Fellowship to E.H.-Y. The German Research Foundation also supported this work under Grant RA969/12–1 to T.R.

## CRediT authorship contribution statement

**Elias Haj-Yehia:** Writing – original draft, Formal analysis, Data curation, Conceptualization. **Raluca I. Mincu:** Writing – review & editing, Data curation, Conceptualization. **Phillip Schulte:** Writing – review & editing, Data curation. **Sebastian Korste:** Writing – review & editing, Visualization, Methodology, Data curation. **Samuel Dautzenberg:** Writing – review & editing, Data curation. **Lars Michel:** Writing – review & editing. **Amir A. Mahabadi:** Writing – review & editing, Data curation. **Tienush Rassaf:** Writing – review & editing, Supervision, Resources. **Matthias Totzeck:** Writing – review & editing, Supervision, Conceptualization.

## Declaration of competing interest

The authors declare that they have no known competing financial interests or personal relationships that could have appeared to influence the work reported in this paper.
