# Supplementary figures and images for: Hemoglobin is associated with cardiotoxicity in melanoma patients without anemia receiving immune checkpoint inhibitor therapy
Source: Int J Cardiol Heart Vasc. 2025 May 8;59:101693. doi: 10.1016/j.ijcha.2025.101693 (PMC12368969; doi:10.1016/j.ijcha.2025.101693)

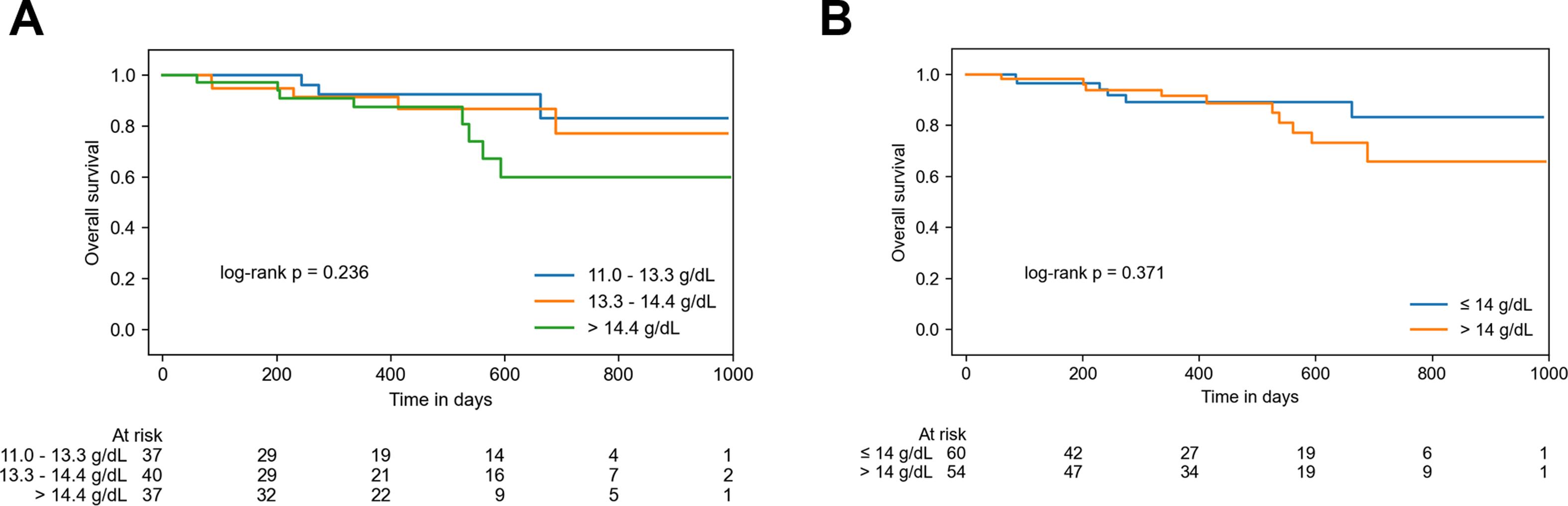

Supplement: Supplementary Fig. 1 [file mmc2.jpg]

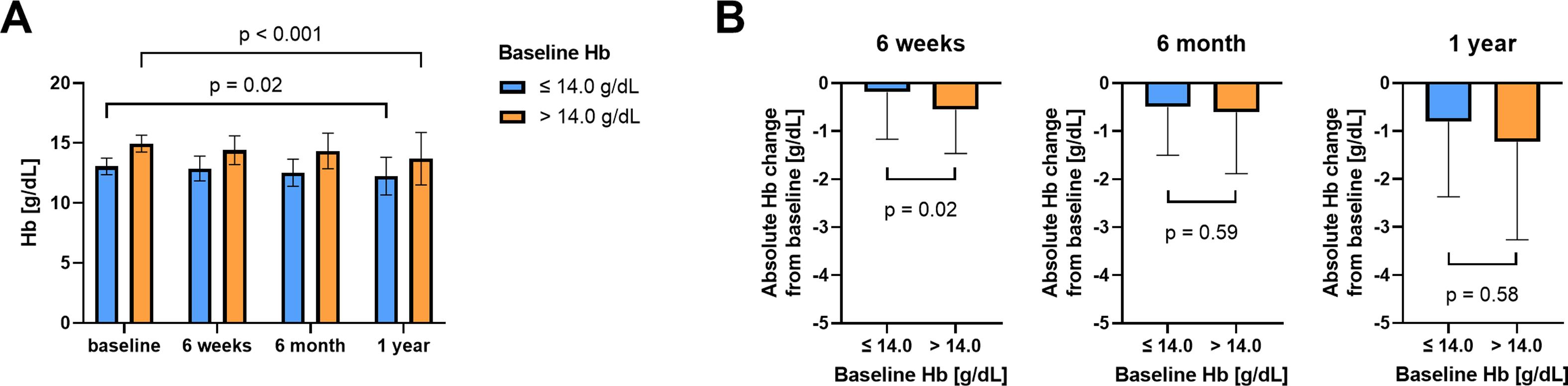

Supplement: Supplementary Fig. 2 [file mmc3.jpg]

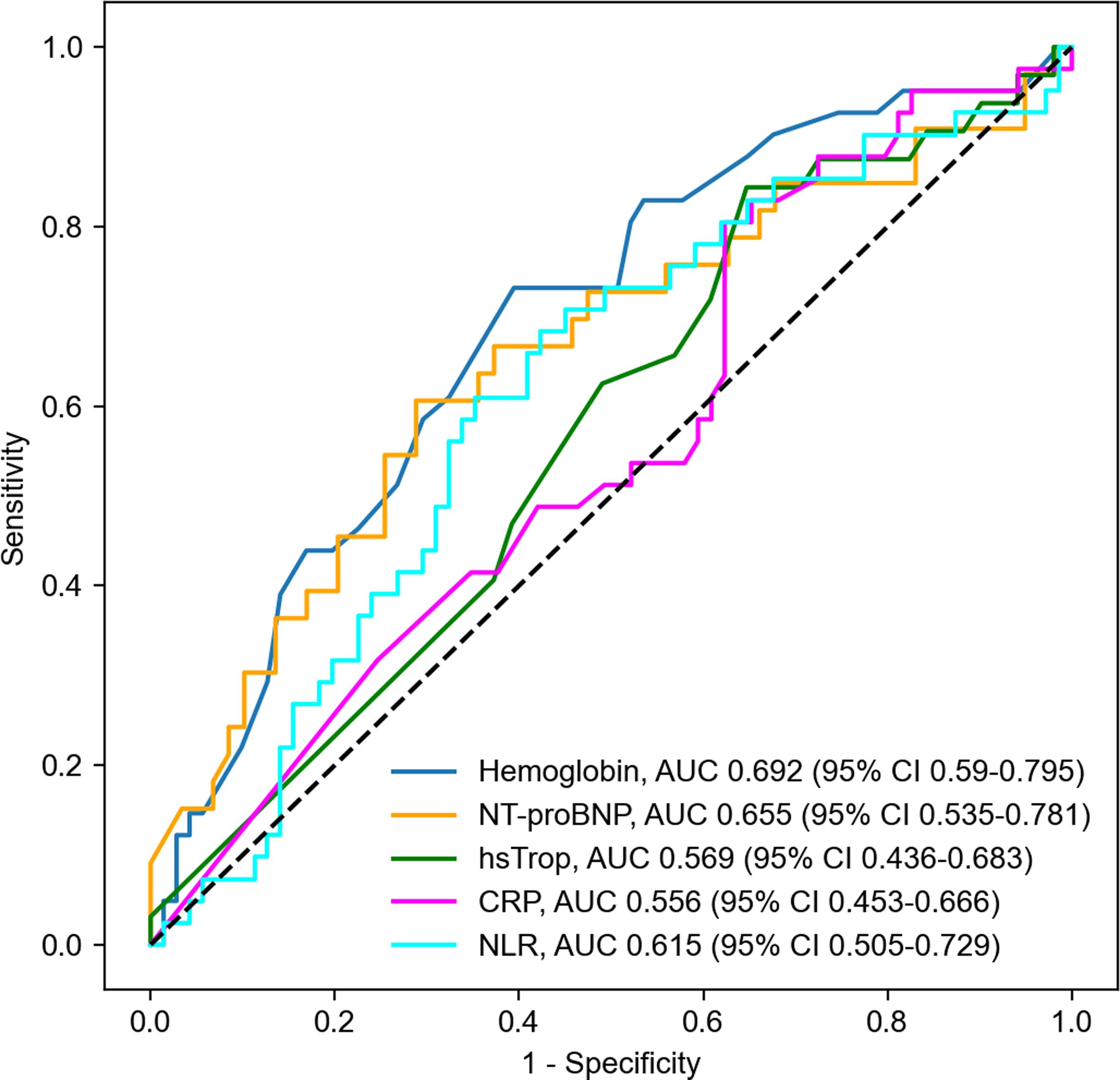

Supplement: Supplementary Fig. 3 [file mmc4.jpg]
