# Supplementary material for: Hemoglobin is associated with cardiotoxicity in melanoma patients without anemia receiving immune checkpoint inhibitor therapy
Source: Int J Cardiol Heart Vasc. 2025 May 8;59:101693. doi: 10.1016/j.ijcha.2025.101693 (PMC12368969; doi:10.1016/j.ijcha.2025.101693)
Supplement: Supplementary Data 1 [file mmc1.docx]

|  |  | **Tertiles of hemoglobin (g/dL)** | | |  |
| --- | --- | --- | --- | --- | --- |
|  | **Total**  **(n = 114)** | **< 13.3**  **(n = 37)** | **13.3 – 14.4**  **(n = 40)** | **> 14.4**  **(n = 37)** | ***p*-value** |
| **ECG parameters, mean ± SD** | | | | | |
| Heart rate (bpm) | 73 ± 12 | 74 ± 13 | 72 ± 11 | 75 ± 12 | 0.74 |
| QRS (ms) | 90.0 ± 14.3 | 87.8 ± 15.0 | 92.2 ± 15.8 | 90.0 ± 11.8 | 0.52 |
| QTc (ms) | 428.3 ± 27.4 | 430.0 ± 27.2 | 426.1 ± 26.2 | 428.7 ± 29.5 | 0.44 |
| **Echocardiography, mean ± SD** | | | | | |
| LV-EF (%) | 60.3 ± 5.1 | 61.7 ± 5.3 | 59.0 ± 4.9 | 60.4 ± 4.7 | 0.35 |
| LV GLS (%) | -20.4 ± 1.7 | -20.4 ± 2.0 | -20.5 ± 1.7 | -20.4 ± 1.5 | 0.95 |
| LAVI (ml/m^2^) | 22.4 ± 7.5 | 22.3 ± 7.7 | 23.6 ± 6.6 | 21.4 ± 8.1 | 0.40 |
| E/E' (ratio) | 7.9 ± 2.0 | 8.1 ± 2.2 | 8.2 ± 2.1 | 7.5 ± 1.6 | 0.58 |
| sPAP (mmHg) | 28.7 ± 6.6 | 29.5 ± 7.0 | 28.1 ± 6.9 | 28.4 ± 5.7 | 0.40 |
| TAPSE (mm) | 27.2 ± 4.6 | 27.8 ± 4.5 | 26.5 ± 5.5 | 27.3 ± 3.6 | 0.79 |

**Supplemental Table 1:** Functional cardiac parameters at baseline

Variables are expressed as mean ± standard deviation (SD). ECG, electrocardiography; bpm, beats per minute; LV-EF, left ventricular ejection fraction; LV GLS, left ventricular global longitudinal strain; LAVI, left atrium volume index; sPAP, systolic pulmonary artery pressure; TAPSE, tricuspid annular plane systolic excursion.

**Supplemental Table 2:** Univariable Cox regression for hemoglobin – subgroups of vascular toxicity and arrhythmia

| **Outcome** | **Number of events** | **Estimated hazard ratio (95% CI)** | ***p*-value** |
| --- | --- | --- | --- |
| **Vascular toxicitiy** |  |  |  |
| Pulmonary embolism | 3 | 0.659 (0.240-1.814) | 0.42 |
| Deep venous thrombosis | 7 | 1.707 (0.942-3.095) | 0.078 |
| Myocardial infarction or stroke | 2 | 3.077 (0.887-10.676) | 0.077 |
| **Arrhythmia** |  |  |  |
| Sinus tachycardia | 10 | 1.364 (0.829-2.246) | 0.22 |
| Sinus bradycardia | 1 | 0.524 (0.083-3.290) | 0.49 |
| Atrial fibrillation | 1 | 0.063 (0.001-3.677) | 0.18 |

CI, confidence interval.

**Supplemental Table 3:** Univariable Cox regression for hemoglobin – subgroups of CTRCD

| **Outcome** | **Number of events** | **Estimated hazard ratio (95% CI)** | ***p*-value** |
| --- | --- | --- | --- |
| Mild CTRCD | 39 | 1.408 (1.086-1.825) | 0.01 |
| LV GLS reduction > 15% | 13 | 1.352 (0.863-2.119) | 0.19 |
| New rise of cardiac biomarkers (NT-proBNP, hsTrop) | 26 | 1.413 (1.030-1.939) | 0.03 |
| Moderate CTRCD | 2 | 1.617 (0.515-5.077) | 0.41 |

CTRCD, cancer therapy-related cardiac dysfunction; CI, confidence interval; LV GLS, left ventricular global longitudinal strain; NT-proBNP, N-terminal prohormone of brain natriuretic peptide, hsTrop, high-sensitive troponin.

|  | **Model S1** | |
| --- | --- | --- |
| **Outcome** | Estimated hazard ratio  (95% CI) | *p*-value |
| Overall CTR-CVT | 1.336  (1.060-1.683) | 0.014 |
| CTRCD | 1.405  (1.077-1.834) | 0.012 |

**Supplemental Table 4:** Multivariable Cox Regression for hemoglobin – adjustment for cardiovascular medication

CI, confidence interval; CTR-CVT, cancer therapy-related cardiovascular toxicity; CTRCD, cancer therapy-related cardiac dysfunction.

**Supplemental Table 5:** Final Cox regression model after backward elimination

|  | **Overall CTR-CVT** | | **CTRCD** | |
| --- | --- | --- | --- | --- |
| **Variable** | Estimated hazard ratio  (95% CI) | *p*-value | Estimated hazard ratio  (95% CI) | *p*-value |
| Hemoglobin | 1.512  (1.084-2.109) | 0.015 | 1.909  (1.182-3.082) | 0.008 |
| Coronary artery disease |  |  | 7,076  (1.401-35.743) | 0.018 |
| Diabetes mellitus | 436  (19-9,648) | < 0.001 | 42,514  (232-7,786,827) | < 0.001 |
| Dyslipidemia |  |  | 0.003  (0.001-0.084) | < 0.001 |
| Atrial fibrillation |  |  | 1,168  (26-52,339) | < 0.001 |
| History of smoking (current or past) |  |  | 0.185  (0.035-0.979) | 0.047 |
| Duration of ICI therapy | 0.996  (0.994-0.999) | 0.003 | 0.993  (0.990-0.997) | < 0.001 |
| LV-EF |  |  | 0.875  (0.794-0.964) | 0.007 |
| hsTrop | 0.989  (0.997-1.001) | 0.068 | 0.956  (0.935-0.978) | < 0.001 |
| CRP | 0.826  (0.727-0.938) | 0.003 | 0.783  (0.664-0.923) | 0.004 |
| eGFR CKD-EPI |  |  | 1.031  (0.997-1.065) | 0.071 |

CI, confidence interval; CTR-CVT, cancer therapy-related cardiovascular toxicity; CTRCD, cancer therapy-related cardiac dysfunction; ICI, immune checkpoint inhibitor; LV-EF, left ventricular ejection fraction; hsTrop, high-sensitive troponin; CRP, C-reactive protein; eGFR CKD-EPI; estimated glomerular filtration rate using the formula of the Chronic Kidney Disease Epidemiology Collaboration.
